# Supplementary material for: MetaRibo-Seq measures translation in microbiomes
Source: Nat Commun. 2020 Jun 29;11:3268. doi: 10.1038/s41467-020-17081-z (PMC7324362; doi:10.1038/s41467-020-17081-z)
Supplement: Supplementary file 10 — Supplementary Data 7 [file 41467_2020_17081_MOESM10_ESM.zip › File2/Confidence_VeryHigh_Taxonomy/1613_out.krona.html]

Javascript must be enabled to view this page.

members
magnitude
magnitudeUnassigned
count
unassigned
taxon
rank

1613\_out

61

superkingdom
2
61

61
1239
phylum

61
186801
class

61
186802
order

1
31979
family

genus
1485
1


SRS052697\_contig\_number\_41771
1896990
1
species

11
1897042
species

SRS014979\_contig\_number\_contig-100\_11441.11442SRS018623\_contig\_number\_1770SRS020233\_contig\_number\_contig-100\_11543.11543SRS022071\_contig\_number\_contig-100\_14236.14236SRS077641\_contig\_number\_contig-100\_26378.26379SRS098571\_contig\_number\_1391SRS1041037\_contig\_number\_contig-100\_2819.2820SRS104975\_contig\_number\_20102SRS142781\_contig\_number\_5562SRS147346\_contig\_number\_60430SRS149181\_contig\_number\_28660

family
47
186806


SRS017191\_contig\_number\_contig-100\_7174.84023SRS022609\_contig\_number\_32192SRS024331\_contig\_number\_15563SRS024663\_contig\_number\_6540
genus
47
4
1730


SRS012849\_contig\_number\_20845SRS013951\_contig\_number\_10568SRS014235\_contig\_number\_5539SRS014287\_contig\_number\_15883SRS014412\_contig\_number\_contig-100\_18023.18024SRS015578\_contig\_number\_16272SRS016629\_contig\_number\_contig-100\_4166.4167SRS019496\_contig\_number\_contig-100\_10883.58609SRS045244\_contig\_number\_contig-100\_21536.21537SRS058723\_contig\_number\_contig-100\_31654.31654SRS063040\_contig\_number\_contig-100\_1930.197589SRS064557\_contig\_number\_4301SRS075773\_contig\_number\_31274SRS104311\_contig\_number\_29942SRS142890\_contig\_number\_28678SRS146812\_contig\_number\_31916SRS148784\_contig\_number\_25865SRS893285\_contig\_number\_3960
species
142586
18


SRS014855\_contig\_number\_5034
species
1
39485

19
1262889
species

SRS014613\_contig\_number\_contig-100\_936.112317SRS015065\_contig\_number\_contig-100\_12218.100922SRS015431\_contig\_number\_contig-100\_27308.114462SRS015890\_contig\_number\_22877SRS018836\_contig\_number\_contig-100\_8732.8733SRS019068\_contig\_number\_20531SRS042284\_contig\_number\_5290SRS052027\_contig\_number\_8408SRS052697\_contig\_number\_contig-100\_2321.164424SRS065504\_contig\_number\_contig-100\_4823.4823SRS065504\_contig\_number\_contig-100\_3201.83425SRS077194\_contig\_number\_27966SRS098514\_contig\_number\_contig-100\_49.3924SRS104485\_contig\_number\_5182SRS105153\_contig\_number\_9649SRS142503\_contig\_number\_28339SRS143598\_contig\_number\_21799SRS147271\_contig\_number\_contig-100\_16601.55473SRS148784\_contig\_number\_7846

1262895
5
species

SRS015431\_contig\_number\_5407SRS015854\_contig\_number\_10643SRS063985\_contig\_number\_contig-100\_3081.131415SRS104400\_contig\_number\_26031SRS104693\_contig\_number\_3396

family
186803
2

genus
2
841

species
301302
1

SRS019068\_contig\_number\_30867

301301
1
species

SRS1055022\_contig\_number\_1104
